# Perioperative cerebral blood flow measured by arterial spin labeling with different postlabeling delay in patients undergoing carotid endarterectomy: a comparison study with CT perfusion

**DOI:** 10.3389/fnins.2023.1200273

**Published:** 2023-09-14

**Authors:** Huimin Xu, Hualu Han, Ying Liu, Ran Huo, Ning Lang, Huishu Yuan, Tao Wang, Xihai Zhao

**Affiliations:** ^1^Department of Radiology, Peking University Third Hospital, Beijing, China; ^2^Department of Biomedical Engineering, Center for Biomedical Imaging Research, School of Medicine, Tsinghua University, Beijing, China; ^3^Department of Neurosurgery, Peking University Third Hospital, Beijing, China

**Keywords:** magnetic resonance imaging, multidetector computed tomography, perfusion, carotid endarterectomy, arterial spin labeling

## Abstract

**Background:**

Arterial spin labeling (ASL) is a non-invasive technique for measuring cerebral perfusion. Its accuracy is affected by the arterial transit time. This study aimed to (1) evaluate the accuracy of ASL in measuring the cerebral perfusion of patients who underwent carotid endarterectomy (CEA) and (2) determine a better postlabeling delay (PLD) for pre- and postoperative perfusion imaging between 1.5 and 2.0 s.

**Methods:**

A total of 24 patients scheduled for CEA due to severe carotid stenosis were included in this study. All patients underwent ASL with two PLDs (1.5 and 2.0 s) and computed tomography perfusion (CTP) before and after surgery. Cerebral blood flow (CBF) values were measured on the registered CBF images of ASL and CTP. The correlation in measuring perioperative relative CBF (rCBF) and difference ratio of CBF (DR_CBF_) between ASL with PLD of 1.5 s (ASL_1.5_) or 2.0 s (ASL_2.0_) and CTP were also determined.

**Results:**

There were no significant statistical differences in preoperative rCBF measurements between ASL_1.5_ and CTP (*p* = 0.17) and between ASL_2.0_ and CTP (*p* = 0.42). Similarly, no significant differences were found in rCBF between ASL_1.5_ and CTP (*p* = 0.59) and between ASL_2.0_ and CTP (*p* = 0.93) after CEA. The DR_CBF_ measured by CTP was found to be marginally lower than that measured by ASL_2.0_1.5_ (*p* = 0.06) and significantly lower than that measured by ASL_1.5_1.5_ (*p* = 0.01), ASL_2.0_2.0_ (*p* = 0.03), and ASL1_.5_2.0_ (*p* = 0.007). There was a strong correlation in measuring perioperative rCBF and DR_CBF_ between ASL and CTP (r = 0.67–0.85, *p* < 0.001). Using CTP as the reference standard, smaller bias can be achieved in measuring rCBF by ASL_2.0_ (−0.02) than ASL_1.5_ (−0.07) before CEA. In addition, the same bias (0.03) was obtained by ASL_2.0_ and ASL_1.5_ after CEA. The bias of ASL_2.0_2.0_ (0.31) and ASL_2.0_1.5_ (0.32) on DR_CBF_ measurement was similar, and both were smaller than that of ASL_1.5_1.5_ (0.60) and ASL_1.5_2.0_ (0.60).

**Conclusion:**

Strong correlation can be found in assessing perioperative cerebral perfusion between ASL and CTP. During perioperative ASL imaging, the PLD of 2.0 s is better than 1.5 s for preoperative scan, and both 1.5 and 2.0 s are suitable for postoperative scan.

## Introduction

Carotid endarterectomy (CEA) is an effective revascularization method for the treatment of carotid artery stenosis (Ferguson et al., 1999; Rothwell et al., 2003). Until now, a number of studies have suggested that cerebral hemodynamic impairment in patients with or without severe carotid artery stenosis might increase the risk of cerebral infarction and hyperperfusion syndrome (CHS) when the cerebral blood flow (CBF) and cerebrovascular reactivity (CVR) are reduced (Hosoda, 2015). Consequently, the assessment of cerebral perfusion before and after CEA in patients with severe carotid artery stenosis is important for making a treatment strategy, monitoring a surgical effect, and predicting adverse outcomes such as CHS (Hosoda, 2015; Yamamoto et al., 2017). Computed tomography perfusion (CTP) is a widely accepted technique for cerebral perfusion evaluation, which can provide relatively accurate quantitative perfusion parameters, such as CBF (Heit and Wintermark, 2016). Nevertheless, CTP is not suitable for some individuals due to the radiation damage and contrast medium injection (Smith et al., 2003).

Arterial spin labeling (ASL), as an emerging non-invasive magnetic resonance imaging technique, is capable of quantifying CBF by using the protons of arterial blood water molecules as endogenous tracers (Detre et al., 1992). However, the accuracy of CBF measurements is affected by the arterial transit time (ATT), which is the transport time from the labeling position to the tissue (Akiyama et al., 2016). The ATT varies among individuals and between healthy and pathological tissues (Alsop et al., 2015; Lindner et al., 2015). Therefore, in order to mitigate ATT errors to the greatest extent, the choice of appropriate postlabeling delay (PLD), the delay time between the end of the pulse train and image acquisition, becomes important for accurate CBF measurements by ASL (MacIntosh et al., 2010; Haga et al., 2016). Recent studies have generally used a single PLD between 1.5 and 2.0 s in ASL scans (MacIntosh et al., 2010; Lin et al., 2018). The fundamental trade-off is that a short PLD does not allow a complete delivery of the labeled blood to the tissue, whereas a long PLD results in strong T1 decay and, therefore, a reduction of signal-to-noise ratio (SNR) (Knutsson et al., 2010; Wang et al., 2012). However, it is still unknown which PLD of ASL in measuring CBF is more suitable for measuring CBF in patients who undergo CEA perioperatively between 1.5 and 2.0 s. According to the International Society for Magnetic Resonance in Medicine Workshop recommendations, a PLD of 2.0 s is suggested for adults (Alsop et al., 2015) but does not clearly indicate whether ASL imaging needs to keep the same PLD before and after CEA operation or whether the PLD needs to be shortened due to the patency of blood vessels after CEA operation.

The goal of this study was to investigate the accuracy of ASL in measuring cerebral perfusion and determine better PLDs for ASL in measuring CBF in patients with severe carotid atherosclerotic stenosis before and after CEA compared with CTP.

## Materials and methods

### Patients

Patients scheduled for CEA with unilateral severe carotid artery stenosis [70–99%, according to the North American Symptomatic Carotid Endarterectomy Trial (NASCET) grading] as determined by CT angiography (CTA) were recruited in this study. The exclusion criteria were as follows: (1) allergic to contrast agents; (2) heart failure; (3) renal dysfunction (glomerular filtration rate < 60 mL/min); (4) contraindications to MR examination; (5) history of carotid endarterectomy or carotid stenting; and (6) intracranial arterial stenosis ≥50%. All the included patients underwent MR imaging and CT scan before and after (within 1 week) CEA. The clinical information including age, gender, history of smoking, hypertension, hyperlipidemia, diabetes, and stroke was collected from the clinical record at baseline.

**Figure 1:**
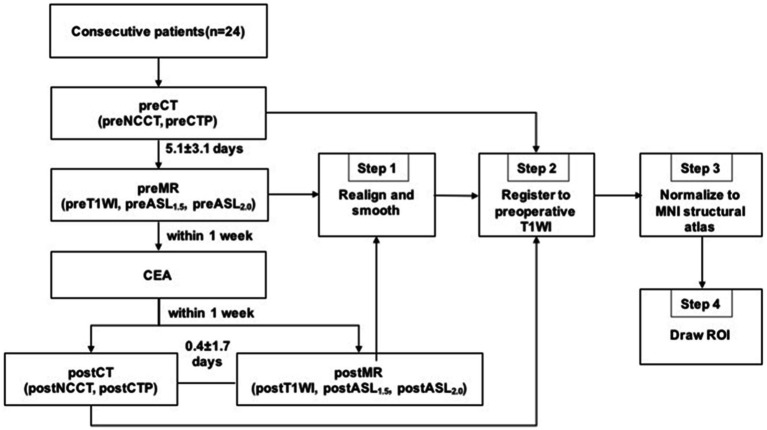
Imaging examinations and image processing flow chart. Pre, preoperative; post, postoperative; CEA, carotid endarterectomy.

The local ethics committee approved the study protocol, and written informed consent was obtained from all patients. A flowchart of the study process is shown in Figure 1.

### CT scan protocol

CT imaging was performed on a 256-row wide-body detector CT scanner (Revolution CT, GE Healthcare, Waukesha, Wisconsin, United States). The scan protocol included non-contrast enhanced CT (NCCT) and CTP. A routine NCCT with a spatial resolution of 0.49× 0.49 × 5 mm^3^ covering the whole brain was performed for registration as described below. After NCCT scan, the whole brain volumetric CTP with 16 cm z-axis coverage was acquired with 0.5 mm slice thickness and 80 kV tube voltage. A bolus of 40 mL non-ionic contrast agent (370 mgI/mL, Omnipaque 350; GE Healthcare, Shanghai, China) was injected intravenously with an automatic injector at a rate of 4.5 mL/s, followed by a 40 mL saline flush at 4.5 mL/s. The CTP protocol was initiated 8 s after contrast agent injection by 10 scans with 100 mAs and a 2 s interval, followed by seven scans with 75 mAs and a 4 s interval. The total scan duration was 56 s and the total absorbed radiation dose was 5.4 mSv.

### MR imaging protocol

MR scan was performed after CT examination (1–7 days before CEA or 2 days after CEA) on a 3.0 T whole-body scanner (Discovery 750, GE Medical Systems) equipped with an eight-channel head coil. The whole brain three-dimensional pulse-continuous ASL (3D-PCASL) was acquired using a 3D spiral fast spin-echo sequence with background suppression for perfusion imaging, and the labeling plane was placed perpendicular to the carotid arteries around C2/C3 (Bouthillier et al., 1996). Other acquisition parameters were as follows: two PLDs, 1.5 and 2.0 s (acquisition times were 3.15 and 3.44 min, respectively); repeat time, 4,632 ms (PLD = 1.5 s) and 4,842 ms (PLD = 2.0 s); echo time, 10.5 ms; field of view, 25 × 25 cm^2^; voxel size, 2 × 2 × 4 mm^3^; labeling duration, 1.5 s. In addition, the structural imaging for registration was a sagittal 3D T1-weighted sequence with the following parameters: TR = 4.9 ms, TE = 2 ms, 15°flip angle, 170 sections, voxel size = 1.0 × 0.9 × 0.9 mm3, FOV = 24 × 24 cm.

### Data processing

**Figure 2:**
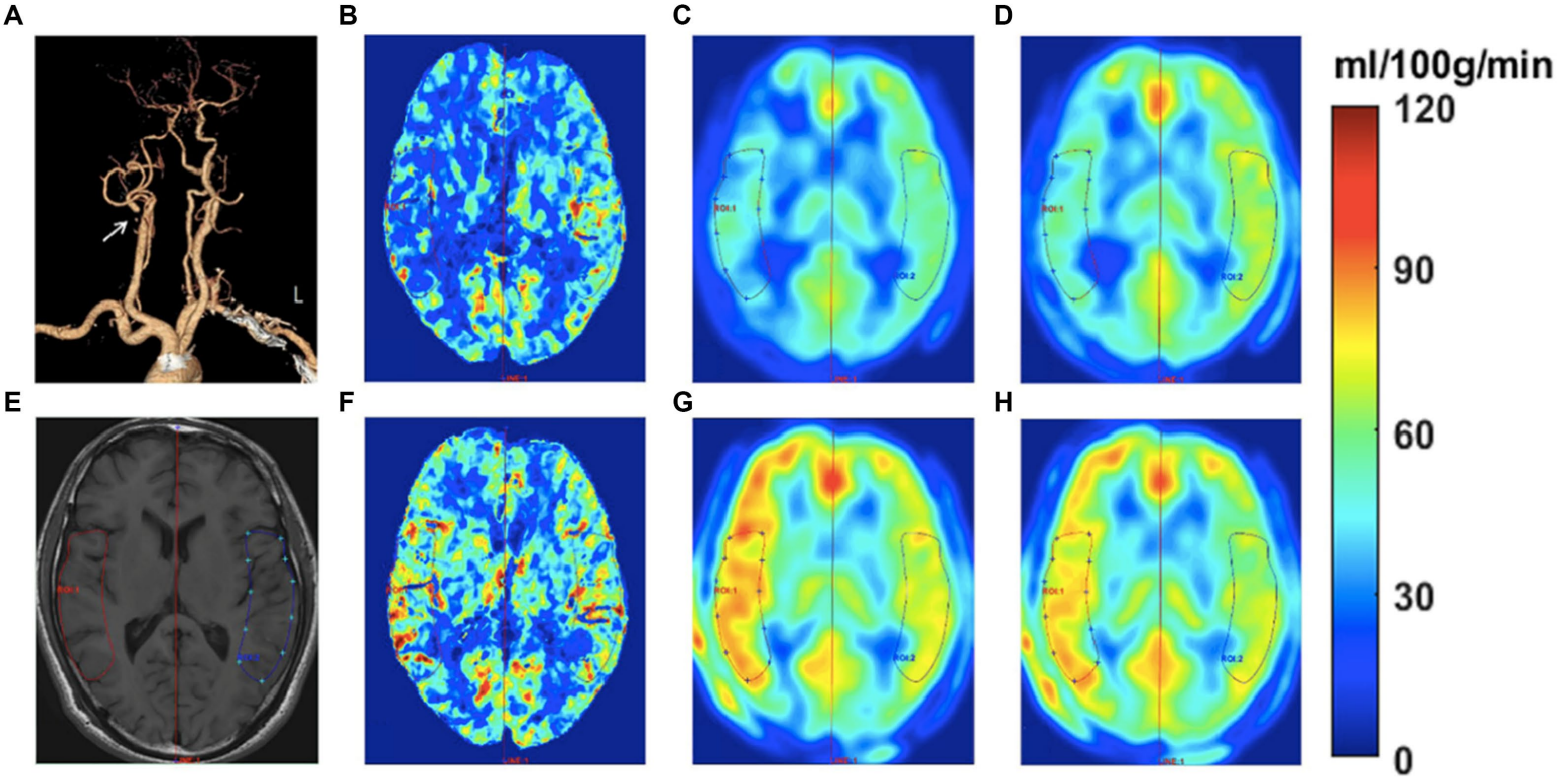
Images of a 58-year-old man with transient ischemic attack (TIA) for half a year. The CTA **(A)** shows occlusive disease at the right proximal internal carotid artery (see white arrow). The ROIs were drawn on the T1W image to measure the CBF at bilateral sides **(E)**. The CBF color-coded maps of preoperative and postoperative CTP, ASL1.5, and ASL2.0 are shown in B-D and F-H, respectively. The perioperative ASL-CBF maps **(C,D,G,H)** were visually comparable with the CTP-CBF maps **(B,F)**. The preoperative ASL-CBF maps **(C,D)** show lower CBF values compared to CTP-CBF **(B)** on the occlusive side. In contrast, the postoperative ASL-CBF maps **(G,H)** show higher CBF values than CTP-CBF **(F)** on the occlusive side.

Image analysis was carried out using Matlab 2016a (MathWorks, Natick, MA) and SPM12 (Wellcome Trust Centre for NeuroImaging, UCL, United Kingdom). The imaging processing included four steps: Step 1: the perioperative MR images including the T1-weighted images and CBF images of ASL with two PLDs were realigned and smoothed using SPM12; Step 2: all perioperative ASL and CTP images were registered to the preoperative T1-weighted MRI. The perioperative CTP images were registered to the T1-weighted image using the whole brain NCCT as an interim template; Step 3: the preoperative T1-weighted image and all of the perfusion images were spatially normalized to Montreal Neurological Institute (MNI) space with a spatial resolution of 2 × 2 × 2 mm^3^; Step 4: the CBF values were obtained from the different perioperative perfusion images. ROIs were drawn manually by two neuroradiologists (both with over 5 years of experience in processing ASL and CTP images) who were blinded to the degree of stenosis or clinical history. The mean time interval between two ROI delineations of intra-observer was 1 month. ROIs were positioned on the T1-weighted image of each patient to ensure that the infarcted and bone tissues were excluded from the ROIs. ROIs representing the middle cerebral artery (MCA) distributions were outlined manually on the three slabs closest to the level of the lateral ventricle on the operative side according to the maps of Damasio (Waaijer et al., 2007). Subsequently, the axis of symmetry was traced on the midline and a mirror image of the ROI was reflected onto the contralateral hemisphere. All six ROIs on the three slabs were then automatically transferred to the remaining registered perfusion images. Thus, the location of ROIs could have the same dimensions and topography in both hemispheres and remain consistent within different perfusion images of the same patient. The procedure for drawing ROIs is shown in Figure 2.

The average values of three ROIs on the one side were considered as the mean CBF value in the MCA territory. Importantly, a non-diffusible tracer was used for CTP, whereas a diffusible tracer was used for ASL. Moreover, brain perfusion measurements are subject to high inter-subject variation and are influenced by physiologic parameters. Therefore, to minimize the variability in absolute quantification of perfusion parameters of ASL and CTP, we used the relative CBF (rCBF) to represent the perioperative cerebral hemodynamics by normalizing the CBF values measured on the surgical side to the values measured on the other side, which was calculated as the surgical hemisphere-to-contralateral hemisphere ratio (rCBF=CBF_sur_/CBF_contra_). In addition, in order to evaluate the changes of CBF on the surgical side after CEA, we calculated the perioperative difference ratio of CBF (DR_CBF_) as the ratio of perioperative difference of CBF values to preoperative CBF values (DR_CBF_ = [CBF_post_-CBF_pre_]/CBF_pre_). The DR_CBF_ of ASL was taken from the following four PLD combinations: ASL_1.5_1.5_: perioperative PLD = 1.5 s; ASL_2.0_2.0_: perioperative PLD = 2.0 s; ASL_2.0_1.5_: preoperative PLD = 2.0 s and postoperative PLD = 1.5 s; and ASL_1.5_2.0_: preoperative PLD = 1.5 s and postoperative PLD = 2.0 s.

### Statistical analyses

Continuous variables were presented as mean value ± standard deviation if normally distributed. Otherwise, variables were presented as median with interquartile range (IQR). The two-way mixed intra-class correlation coefficient (ICC) was used to assess the inter-observer and intra-observer agreements for ROI drawing. ICC values less than 0.5, between 0.5 and 0.75, between 0.75 and 0.9, and greater than 0.90 are indicative of poor, moderate, good, and excellent reliability, respectively (Koo and Li, 2016). The perioperative rCBF and DR_CBF_ were compared between ASL_1.5_ (ASL with PLD of 1.5 s) or ASL_2.0_ (ASL with PLD of 2.0 s) and CTP using the Wilcoxon signed rank test. The correlation of perioperative rCBF and DR_CBF_ between ASL_1.5_ or ASL_2.0_ and CTP was analyzed using Pearson’s correlation coefficients. Correlation between ASL and CTP was expressed as *r*-value, and *r*-values were defined as follows: 0–0.20, no correlation; 0.21–0.40, weak correlation; 0.41–0.60, moderate correlation; 0.61–0.80, strong correlation; and greater than 0.81, very strong correlation (Landis and Koch, 1977). The Bland–Altman analysis was used to assess the bias between ASL_1.5_ or ASL_2.0_ and CTP in measuring perioperative rCBF and DR_CBF_. A *p* < 0.05 (two-sided) was considered to be statistically significant. All statistical analyses were performed using the SPSS 24.0 software (SPSS, Chicago, IL).

## Results

A total of 27 patients were enrolled in this study from December 2017 to October 2018. Three patients were excluded from statistical analysis due to severe motion artifacts (*n* = 2) and failure to co-registration (*n* = 1). The clinical information of the remaining 24 patients is summarized in Table 1. Of the remaining 24 patients, the mean age was 64.6 ± 7.5 years old and 21 were male patients. Among these patients, 7 (29%) had a history of smoking, 13 (54%) had hypertension, 8 (33%) had hyperlipidemia, and 6 (25%) had diabetes. The mean luminal stenosis of carotid arteries at the surgical site was 84.6% ± 10.7%. The mean time interval between CT and MR imaging before and after CEA was 5.1 ± 3.1 days and 0.4 ± 1.7 days, respectively. During the time interval of CT and MR imaging, no intervention was performed, and no new symptoms were developed.

**Table 1:** Baseline characteristics of patients (*n* = 24).

	Mean ± SD, or n (%)
Age, years	64.6 ± 7.5
Sex, male	21 (88)
Smoking	7 (29)
Hypertension	13 (54)
Hyperlipidemia	8 (33)
Diabetes	6 (25)
Luminal stenosis, %	
Surgical side	84.6 ± 10.7
Contralateral side	31.4 ± 15.7
Time interval between CT and MR scan	
Pre-CEA scan, days	5.1 ± 3.1
Post-CEA scan, days	0.4 ± 1.7
Symptoms	19 (79)
Stroke	8 (33)
TIA	11 (46)

CEA, carotid endarterectomy; TIA, transient ischemic attack.

### Inter-observer and intra-observer agreements for ROI drawing

There were good-to-excellent agreements between two readers and a single reader; the results are shown in Table 2.

**Table 2:** Agreements of inter-observer and intra-observer for ROI drawing.

	Intra-observer agreement		Inter-observer agreement	
	ICC	*p*-value	ICC	*p*-value
Preoperative rCBF of ASL_1.5_	0.992	0.001	0.989	<0.001
Preoperative rCBF of ASL_2.0_	0.989	0.001	0.992	<0.001
Preoperative rCBF of CTP	0.931	0.001	0.87	<0.001
Postoperative rCBF of ASL_1.5_	0.993	0.001	0.99	<0.001
Postoperative rCBF of ASL_2.0_	0.99	0.001	0.988	<0.001
Postoperative rCBF of CTP	0.924	0.001	0.798	<0.001
DR_CBF_ of ASL_1.5_1.5_	0.954	0.001	0.983	<0.001
DR_CBF_ of ASL_1.5_2.0_	0.963	0.001	0.986	<0.001
DR_CBF_ of ASL_2.0_1.5_	0.965	0.001	0.964	<0.001
DR_CBF_ of ASL_2.0_2.0_	0.983	0.001	0.947	<0.001
DR_CBF_ of CTP	0.853	0.001	0.841	<0.001

CTP, CT perfusion; rCBF, relative cerebral blood flow; DR_CBF_, difference ratio of cerebral blood flow; ASL_1.5_1.5_, ASL with perioperative PLD = 1.5 s; ASL_2.0_2.0_, ASL with perioperative PLD = 2.0 s; ASL_2.0_1.5_, ASL with preoperative PLD = 2.0 s and postoperative PLD = 1.5 s; ASL_1.5_2.0_, ASL with preoperative PLD = 1.5 s and postoperative PLD = 2.0 s; ICC, intra-class correlation coefficient.

### Comparison of measurements between ASL and CTP

The Wilcoxon signed rank test demonstrated that there were no significant statistical differences in preoperative rCBF measurements between ASL_1.5_ and CTP (0.98 [IQR, 0.68–1.05] vs. 0.95 [IQR, 0.87–1.03], *p* = 0.17) and between ASL_2.0_ and CTP (1.01 [IQR, 0.71–1.05] vs. 0.95 [IQR, 0.87–1.03], *p* = 0.42). Similarly, no significant differences were found in rCBF between ASL_1.5_ and CTP (1.03 [IQR, 0.90–1.11] vs. 1.04 [IQR, 0.97–1.11], *p* = 0.59) and between ASL_2.0_ and CTP (1.02 [IQR, 0.96–1.09] vs. 1.04 [IQR, 0.97–1.11], *p* = 0.93) after CEA. Table 3 shows the comparison results on DR_CBF_ between CTP and ASL with different combinations of PLDs. The DR_CBF_ measured by CTP was found to be marginally lower than that measured by ASL_2.0_1.5_ (*p* = 0.06) and significantly lower than that measured by ASL_1.5_1.5_ (*p* = 0.01), ASL_2.0_2.0_ (*p* = 0.03), and ASL_1.5_2.0_ (*p* = 0.007).

**Table 3:** Comparison of DR_CBF_ between CTP and ASL with four different PLD combinations.

	CTP	ASL_1.5_1.5_	ASL_2.0_2.0_	ASL_2.0_1.5_	ASL_1.5_2.0_
Median	0.17	0.26	0.15	0.17	0.25
IQR	−0.05 to 0.33	−0.02 to 0.86	0.02–0.59	−0.03 to 0.73	0.02–0.73
*p*-value	–	0.01	0.03	0.06	0.007

CTP, CT perfusion; ASL_1.5_1.5_, ASL with perioperative PLD = 1.5 s; ASL_2.0_2.0_, ASL with perioperative PLD = 2.0 s; ASL_2.0_1.5_, ASL with preoperative PLD = 2.0 s and postoperative PLD = 1.5 s; ASL_1.5_2.0_, ASL with preoperative PLD = 1.5 s and postoperative PLD = 2.0 s; IQR, interquartile range.

### Correlation of measurements between ASL and CTP

**Figure 3:**
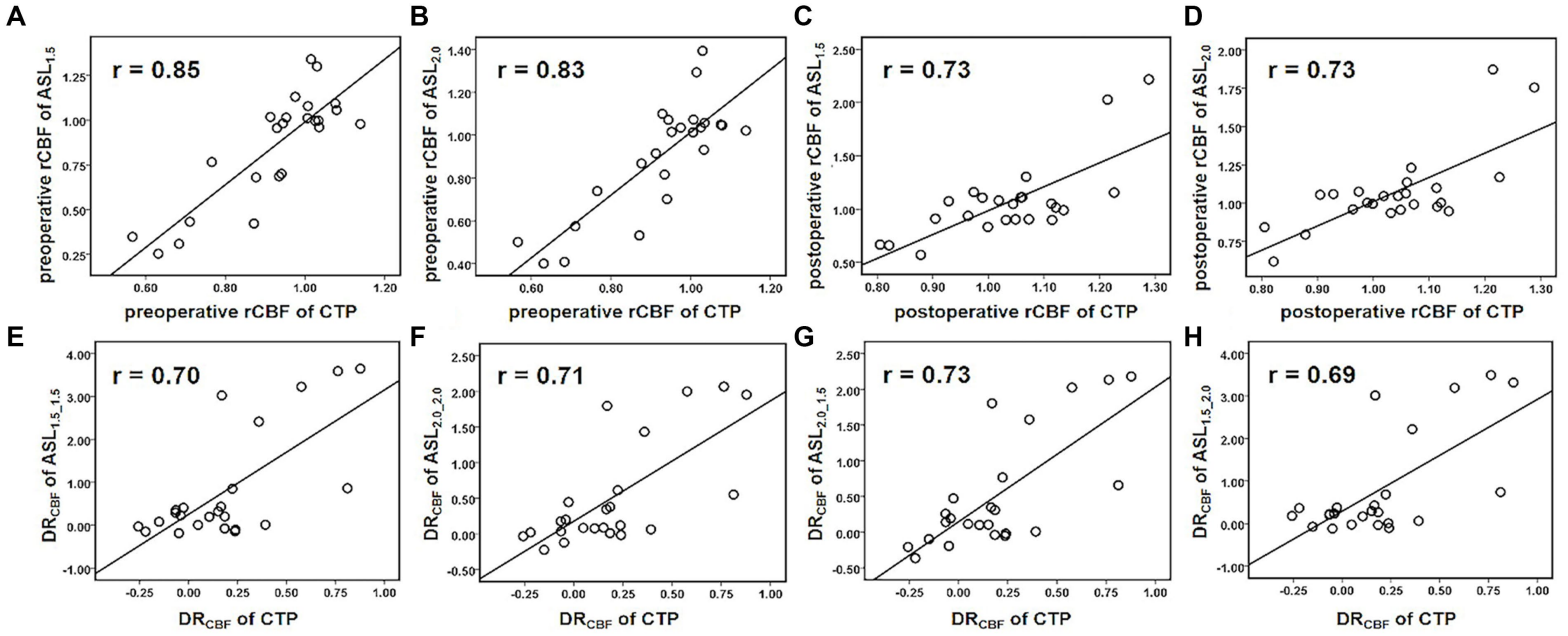
Scatterplots for Pearson’s correlation coefficients between ASL and CTP in measuring perioperative rCBF and DR_CBF_. Both ASL1.5 **(A)** and ASL2.0 **(B)** showed a very strong correlation with CTP in measuring preoperative rCBF. **(C–H)** Show strong correlations between ASL1.5 or ASL2.0 and CTP in measuring postoperative rCBF **(C,D)**, preoperative **(E,F)**, and postoperative **(G,H)** DRCBF, and the measurements deviate with its increase.

For the preoperative rCBF, there was a very strong correlation between CTP and ASL (PLD = 1.5 s: *r* = 0.85, *p* < 0.001; PLD = 2.0 s: *r* = 0.83, *p* < 0.001, Figures 3A,B). After CEA, strong correlation can be found in rCBF between CTP and ASL (PLD = 1.5 s: *r* = 0.73, *p* < 0.01; PLD = 2.0 s: *r* = 0.73, *p* < 0.01, Figures 3C,D). In addition, DR_CBF_ measured by CTP was significantly associated with that measured by ASL with different combinations of PLDs (*r* = 0.69–0.73, *p* < 0.01, Figures 3E–H).

**Figure 4:**
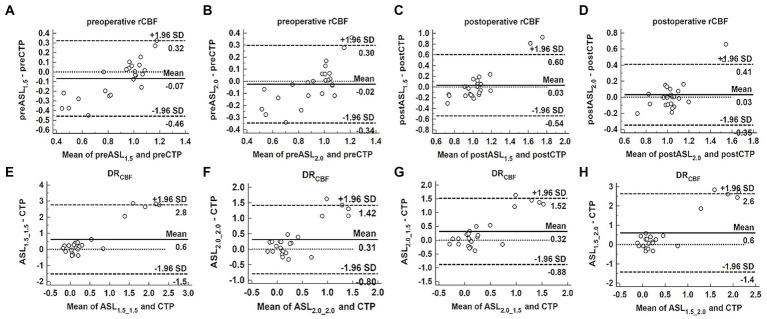
Bland–Altman analysis to assess the bias between ASL and CTP in measuring perioperative rCBF and DR_CBF_. Bland–Altman plots of the difference of ASL and CTP (y-axis) against the mean of ASL and CTP (x-axis) in measuring perioperative rCBF and DR_CBF_, with the mean absolute difference (bias; unbroken lines), the 95% confidence interval of the mean difference (limit of agreement; dashed lines) and the line of no difference (0). Both ASL_1.5_ **(A)** and ASL_2.0_ **(B)** showed the bias in measuring preoperative rCBF, and the bias increases as mean deviates. **(C–H)** Show the bias between ASL_1.5_ or ASL_2.0_ and CTP in measuring postoperative rCBF **(C,D)**, preoperative **(E,F),** and postoperative **(G,H)** DR_CBF_, and the bias becomes larger as mean increases.

The Bland–Altman analysis revealed that the preoperative rCBF measured by ASL was lower than that measured by CTP and the bias of ASL_1.5_ (−0.07) was greater than that of ASL_2.0_ (−0.02) (Figures 4A,B). In contrast, the postoperative rCBF measured by ASL was greater than that measured by CTP, and the bias of ASL_1.5_ (0.03) was the same as of ASL_2.0_ (0.03) (Figures 4C,D). DR_CBF_ measured by ASL was greater than that measured by CTP and the bias of ASL_2.0_2.0_ (0.31) and ASL_2.0_1.5_ (0.32) was smaller than that of ASL_1.5_1.5_ (0.60) and ASL_1.5_2.0_ (0.60) (Figures 4E–H).

## Discussion

This study examined the correlation in measuring perioperative cerebral perfusion between ASL with different PLDs (1.5 and 2.0 s) and CTP in patients with severe carotid atherosclerotic stenosis who underwent CEA. Before and after CEA, we found a strong correlation in measuring rCBF and DR_CBF_ between ASL and CTP. When CTP was considered as a reference, smaller bias can be achieved in measuring rCBF by ASL with PLD of 2.0 s than that of 1.5 s before CEA, and similar bias was obtained by ASL with PLD of 2.0 s and that of 1.5 s after CEA. Our findings suggest that ASL might be an alternative non-contrast enhanced imaging tool for measuring cerebral perfusion and the PLD of 2.0 s is better than 1.5 s in preoperative measurement, and both PLDs 1.5 and 2.0 s can be utilized for postoperative measurement.

In our study, we found a strong correlation in measuring the perioperative rCBF between ASL and CTP, which suggested that ASL might be an alternative non-contrast enhanced approach in assessing cerebral perfusion. Similar findings were reported in previous studies that compared ASL with various invasive and non-invasive methods in terms of CBF measurements (Knutsson et al., 2010; Wang et al., 2014; Xu et al., 2021). A study about Moyamoya disease confirmed that multi-delay ASL with four PLDs (1.5/2/2.5/3 s) improves the CBF accuracy compared with single PLD (2 s); in addition, moderate correlation was found between CBF calculated by multi-delay ASL and CTP (*r* = 0.604) (Wang et al., 2014). Furthermore, moderate-to-high positive associations between ASL-CBF and CTP-CBF were acquired in the gray matter, white matter, and whole brain of the enrolled patients with ischemic stroke (all *p* < 0.005), and the average Pearson correlation coefficients were 0.647, 0.585, and 0.646, respectively (Xu et al., 2021).

In this study, we found that rCBF measured by ASL was slightly smaller and greater than that measured by CTP before and after CEA, respectively. The bias in this study was not significant, which suggests that the observed differences may be purely accidental; however, these data were consistent with previous studies. Tian et al. found that ASL tended to overestimate the perfusion deficit in patients with severe MCA stenosis as compared with CTP, but there were no significant differences between ASL and CTP for those with mild and moderate MCA stenosis (Tian et al., 2018). Furthermore, Koziak et al. found that shorter PLD time (1.2 s) might lead to the overestimation of CBF due to substantial intravascular signal (Koziak et al., 2008). Moreover, Haga et al. found that PLD with 1.5 s would lead to an overestimation of the CBF due to the improvement in anterograde ICA perfusion after CEA (Haga et al., 2019). Several hypotheses have been proposed to explain these phenomena: (1) preoperatively, ATT might be prolonged due to the severe stenosis of the feeding arteries and the formation of collateral blood flow, leading to a loss of perfusion signal in which the labeled blood does not arrive between the time of labeling and image acquisition, and finally results in artificially low CBF values (Calamante et al., 1999). (2) After CEA, acceleration of the anterograde flow in the internal carotid artery may shorten the ATT and lead to an overestimation of the CBF (Shimogawa et al., 2016).

Using CTP as the reference, we found that the preoperative rCBF measured by ASL_2.0_ has a better correlation with CTP than ASL_1.5_. This indicates that ASL with PLD of 2.0 s might be more suitable for patients with severe carotid stenosis before CEA, which is consistent with previous studies (Knutsson et al., 2010; Qiu et al., 2012; Alsop et al., 2015). Longer PLD values allow enough time for the label to reach the tissue, which in turn sustains the accuracy of CBF measurement by overcoming the reduction of SNR caused by time extension. Our study also demonstrated that both PLDs of 1.5 and 2.0 s might be appropriate for evaluating postoperative rCBF because of the same bias compared with CTP. This may be due to that perfusion imaging with shorter PLD (ASL_1.5_) led to a strong T1 signal, whereas longer PLD (ASL_2.0_) allowed more of the labeled water to reach the tissue.

In our study, the changes in cerebral blood flow after CEA (DR_CBF_) measured by ASL were significantly higher than that measured by CTP, suggesting that ASL may overestimate the changes in CBF after CEA. This may be due to the effect of ATT, which leads to the underestimation of CBF values before CEA and the overestimation of CBF values after CEA. In order to reduce the impact of ATT on the accuracy of ASL on DR_CBF_ calculations, we attempted to find better perioperative PLD combinations. We found that the bias of ASL_2.0_2.0_ and ASL_2.0_1.5_ was nearly same, and both were smaller than that of ASL_1.5_1.5_ and ASL_1.5_2.0_. These findings further compel the evidence that PLD of 2.0 s can be used for preoperative ASL imaging, whereas both PLD of 2.0 s and PLD of 1.5 s might be appropriate for postoperative ASL imaging. DR_CBF_ can assist to assess the surgical efficacy and diagnose CHS after CEA. CHS is defined as an increase in postoperative CBF of at least 100% measured by different perfusion imaging methods, such as single photon emission computed tomography (SPECT), CTP, or ASL (DR_CBF_ ≥ 100%) (Piepgras et al., 1988; Ogasawara et al., 2003; Yoshie et al., 2016; Lin et al., 2019). Among the 24 patients in our group, the largest DR_CBF_ measured by CTP was 87.8%, which was 2–3.5-fold of that measured by ASL with different PLD combinations. Apparently, ASL overestimates the measurement of DR_CBF_ compared with CTP and the criteria for diagnosing CHS by each perfusion imaging method might need to be re-modified.

This study has several limitations. First, due to the limited number of patients undergoing CEA surgery in our hospital, the number of cases was small, which could introduce sample bias; thus, a larger sample size would be required in future studies. Second, ASL and CTP were not performed on the same day. However, there were no interventions or cerebrovascular symptoms between the two examinations in any of the patients. Third, we only chose the MCA territory as the representative territory of blood supply from the ipsilateral internal carotid artery. This is mainly due to the tissue supplied by the anterior cerebral arteries (ACA), which can receive flow from either side of the carotid arteries due to the common variation of ACA (Tavares et al., 2010). Furthermore, accurate drawing of the MCA territory was challenging. Even though the consistency of ROI size and shape on both sides of MCA territory was guaranteed, potential bias may be introduced by including portions of the temporal and occipital lobes in the ROI. Finally, since the scan requires long time, we only used two PLDs (1.5 and 2.0 s), while previous studies have reported using PLDs that ranged from 1.5 to 3 s for their multi-PLD experiments (Waaijer et al., 2007; Akiyama et al., 2016; Hara et al., 2017). The use of two PLDs of 1.5 and 2.0 s was based on The International Society for Magnetic Resonance in Medicine Workshop recommendations, as a trade-off between maintaining adequate diagnostic quality (SNR) and allowing sufficient delay for visualizing tissue perfusion in clinical MRI machines (Calamante et al., 1999; Iwanaga et al., 2014; Alsop et al., 2015). It is advisable to determine the optimal PLD for ASL in patients who underwent CEA using more PLDs.

In conclusion, a strong correlation can be found in assessing perioperative cerebral perfusion between ASL and CTP. During perioperative ASL imaging, the PLD of 2.0 s is better than 1.5 s for preoperative scan, and both 1.5 and 2.0 s are suitable for postoperative scan.

## Data availability statement

The original contributions presented in the study are included in the article/Supplementary material, further inquiries can be directed to the corresponding authors.

## Ethics statement

The studies involving human participants were reviewed and approved by Ethics Committee of Peking University Third Hospital. The patients/participants provided their written informed consent to participate in this study.

## Author contributions

HX was responsible for drafting the manuscript, as well as the acquisition, analysis, and interpretation of data. HH, RH, and YL helped to collect, analyze, and interpret the data. NL and XZ helped to revise this article. HY and TW contributed to the conception and design of the current study. All authors read and approved the final manuscript.

## Funding

This study was funded by the grants of Peking University Third Hospital (BYSY2015013), the National Natural Science Foundation of China (81771825), the Beijing Municipal Science and Technology Commission (D17110003017003), and the Ministry of Science and Technology of China (2017YFC1307904).

## Conflict of interest

The authors declare that the research was conducted in the absence of any commercial or financial relationships that could be construed as a potential conflict of interest.

## Publisher’s note

All claims expressed in this article are solely those of the authors and do not necessarily represent those of their affiliated organizations, or those of the publisher, the editors and the reviewers. Any product that may be evaluated in this article, or claim that may be made by its manufacturer, is not guaranteed or endorsed by the publisher.

## Abbreviations

ASL: arterial spin labeling
CEA: carotid endarterectomy
PLD: postlabeling delay
CTP: computed tomography perfusion
CBF: cerebral blood flow
rCBF: relative cerebral blood flow
DR_CBF_: difference ratio of CBF
CHS: cerebral hyperperfusion syndrome
ATT: arterial transit time
SNR: signal-to-noise ratio
NCCT: non-contrast enhanced CT
MCA: middle cerebral artery
